# Temperature Effects on Pea Plants Probed by Simultaneous Measurements of the Kinetics of Prompt Fluorescence, Delayed Fluorescence and Modulated 820 nm Reflection

**DOI:** 10.1371/journal.pone.0059433

**Published:** 2013-03-19

**Authors:** Abdallah Oukarroum, Vasilij Goltsev, Reto J. Strasser

**Affiliations:** 1 Bioenergetics Laboratory, University of Geneva, Geneva, Switzerland; 2 Department of Chemistry and Biochemistry, University of Quebec in Montréal, Montréal, Quebec, Canada; 3 Department of Biophysics and Radiobiology, University of Sofia, Sofia, Bulgaria; University of Hyderabad, India

## Abstract

Simultaneous *in vivo* measurements of prompt fluorescence (PF), delayed fluorescence (DF) and 820-nm reflection (MR) were made to probe response of pea leaves to 40 s incubation at high temperatures (25–50°C). We interpret our observation to suggest that heat treatment provokes an inhibition of electron donation by the oxygen evolving complex. DF, in a time range from several microseconds to milliseconds, has been thought to reflect recombination, in the dark, between the reduced primary electron acceptor Q_A_^–^ and the oxidized donor (P680^+^) of photosystem II (PSII). The lower electron transport rate through PSII after 45 and 50°C incubation also changed DF induction. We observed a decrease in the amplitude of the DF curve and a change in its shape and in its decay. Acceleration of P700^+^ and PC^+^ re-reduction was induced by 45°C treatment but after 50°C its reduction was slower, indicating inhibition of photosystem I. We suggest that simultaneous PF, MR and DF might provide useful information on assessing the degree of plant tolerance to different environmental stresses.

## Introduction

Simultaneous chlorophyll (Chl) *a* fluorescence and 820-nm transmission measurements have provided strong experimental evidence that the three phases (i.e. O-J, J-I and I-P) of the prompt fluorescence rise OJIP [1] reflect three different reduction processes of the electron transport chain [2], [3], [4]. Following a dark-to-light transition of a photosynthetic sample, prompt fluorescence (PF) is emitted and during light-to-dark transition, delayed fluorescence emission (DF) is detected [4], [5]. DF was discovered by Strehler and Arnold [6]. It is mainly emitted from photosystem II (PS II), and photosystem I (PS I) contributes very little to the DF emission [7]. PF depends on the redox state of the PS II reaction centers (RC); however the DF in a time range from several microseconds to milliseconds, after light excitation, has been thought to reflect the recombination, in the dark, between the reduced primary electron acceptor Q_A_^–^ and the oxidized donor (P680^+^) of PSII that are formed after light-induced charge separation [8]. DF has components that decay in very different time domains. From microseconds to milliseconds, DF has been thought to reflect the recombination between the reduced electron acceptor Q_A_^–^ and the oxidized secondary electron donor, Z^+^, of PSII [9]. In the second time range, DF is associated with the recombination of S_2_ and S_3_ states of the oxygen-evolving complex (OEC) with Q_A_^–^ and Q_B_^–^ [10]–[12].

Among others, Grabolle and Dau [13] have reported that the emission spectra of the prompt chlorophyll fluorescence and delayed fluorescence emission in PII membrane particles of spinach are essentially identical. The intensity of DF depends directly on the rate of backward electron transport reactions in the RC of PSII [7], [14]. The shape of the DF induction curve depends on the sample type and its physiological state [9], [15], [16]; further, DF induction curve depends on the kinetic components of DF being measured [17].

High temperature effects on PSII using PF have been measured by many authors [18]–[21]. It is known that the exposure of plants to high temperatures leads to a loss of the manganese cluster [18], [22], [23], which leads to changes in the structure and function of PSII [24], [25]. The manganese cluster of PSII has been identified as the most heat sensitive component of the photosynthetic electron transport chain [18]. One of the earliest events that take place due to heat stress is the loss of grana stacking following dissociation of peripheral light-harvesting complexes from the core complex [26], [27].

At elevated temperatures, changes in lipid–protein interactions have been associated with increased lipid fluidity of the thylakoid membranes [28] and a close relationship between the physical state of membrane lipids and delayed chlorophyll fluorescence intensity have been observed [29]. In another study, lipid unsaturation was found to exert a strong effect on the delayed fluorescence [30]. Simultaneous measurements of the kinetics of PF, and transmission at 820 nm changes at higher temperature have been used to monitor heat treatment (48°C) on electron flow through PSI in pea plants [31]; that in heat-treated samples the electrons responsible for P700^+^ and PC^+^ reduction arrived much more slowly than in the control samples.

In this study heat-stress-induced changes in PSII photochemistry of pea plants was obtained *in vivo*, using three signals- PF, DF and modulated 820 nm reflection (MR), measured simultaneously. We have shown that simultaneous measurement of PF, DF and MR is an important tool to characterize the effect of high temperature on intact photosynthetic systems and can be used as a tool to monitor these changes induced in the photosynthetic membranes.

## Materials and Methods

### Plants and Heat Treatment

Pea plants (*Pisum sativum* L. cv. Ambassador) were grown in the greenhouse with day/night air temperature of 25/18°C, under long-day conditions (16 h light, 8 h dark). The intensity of light used during growth of the plants was 120 µmol photons m^−2^ s^−1^; sometimes, additional light was given, if needed (OSRAM HQIT 400 W lamps were used). The plants were grown in plastic pots (4 l) containing commercial peat soil.

Two week grown pea plants were kept in dark for at least 1 h. Mature and detached leaves were used in this study. The leaves were submerged in water for 40 s at various temperatures (25, 30, 35, 40, 45 and 50°C) in the dark, and then the kinetics of prompt fluorescence, delayed fluorescence and modulated 820 nm reflection were measured simultaneously after 5 min relaxation at room temperature. Five different leaves were measured for each temperature.

### The Multifunctional Plant Efficiency Analyser M-PEA

In the M-PEA instrument emitter wavelength ranges are: (1) 627±10 nm, for the actinic light LED; (2) 820±25 nm, for the modulated light LED, and (3) 735±15 nm, for the far-red light LED; the latter uses a RG9 long pass filter to remove any visible light component (see [5], [16], and Kalaji et al. 2012). High quality optical band pass filters were used for the protection of the detectors of prompt and delayed fluorescence (730±15 nm) and modulated reflection (820±20 nm). The LED emitting in the far-red (735±15 nm; 1000 µmol photons m^−2^ s^−1^ at 100%) was used when fast re-opening of PSII reaction centers was required (e.g., for samples being at the light-adapted state). The actinic light LED is built into the centre of the optical sensor unit and focused onto the sample surface to provide homogeneous illumination over the exposed circular area (2 mm diameter), with an intensity of 5000 µmol photons m^−2^ s^−1^ at 100%. The other emitters and detectors are built on the periphery of the unit. The data acquisition for the three signals, PF and MR in the light, and DF in the dark, is every 0.01 ms in the digitalization range 1 (0.01–0.3 ms), every 0.1 ms in range 2 (0.3–3 ms), every 1 ms in range 3 (3–30 ms), and then decreases until range 7 (30–300 s), where the data acquisition is every 10 s.

During one measure, the measuring cycle includes light and dark periods in the ratio of 3∶1. The duration of the cycle is increased during the induction in sequence: 400 µs, 4, 10, 100 ms, 1 and 10 s (a schematic representation of the timing protocol for simultaneous recording of prompt and delayed chlorophyll fluorescence was shown in [32]). During the cycle, the PF is measured when the actinic light is on and DF is recorded when the light is off.

The calculated ratio MR/MR_0_ (MR_0_ is the value at the onset of the actinic) is complementary of the fraction (*I*_abs_/*I*_inc_)_820 nm_ of incident light flux (*I*_inc_) that is absorbed (*I*_abs_) by the sample (at 820 nm). A decrease in the amplitude of MR/MR_0_ is equivalent to an increase of (I_abs_/I_inc_)_820 nm_; the latter is associated with a decrease of (*I*_abs_/*I*_inc_)_700 nm_ (photobleaching, at about 700 nm). A decrease of (*I*_abs_/*I*_inc_)_700 nm_ corresponds to an increase in the concentration of oxidized states of PSI reaction center (P700^+^) and plastocyanin (PC^+^); accordingly, an MR/MR_0_ increase indicates P700^+^ and PC^+^ reduction [2].

### Simultaneous Measurements of the Kinetics of Prompt Fluorescence, Delayed Fluorescence and Modulated 820 nm Reflection

Illumination of pea leaves exposed to 25°C exhibits a polyphasic PF rise. The fluorescence rise up to the J-step provides information about single turnover events of the primary reactions of photochemistry, mainly Q_A_ reduction [1]. During the time interval from 2 to ∼200 ms, multiple charge separation occurs and the redox components of the electron transport chain become reduced (see [4] for review). The I–P phase is related to PSI activity [33], [34], [35]. For modulated reflection signals the first reliable MR measurement was at 0.3 ms. The MR decrease exhibits photoinduced oxidation of P700 and accumulation of P700^+^ and PC^+^ until about 20 ms, this accumulation is in the range of the J–I phase of PF. Subsequently, the MR increase exhibits re-reduction of both P700^+^ and PC^+^ by the intersystem electrons in the range of the I–P phase of PF [2].

**Figure 1:**
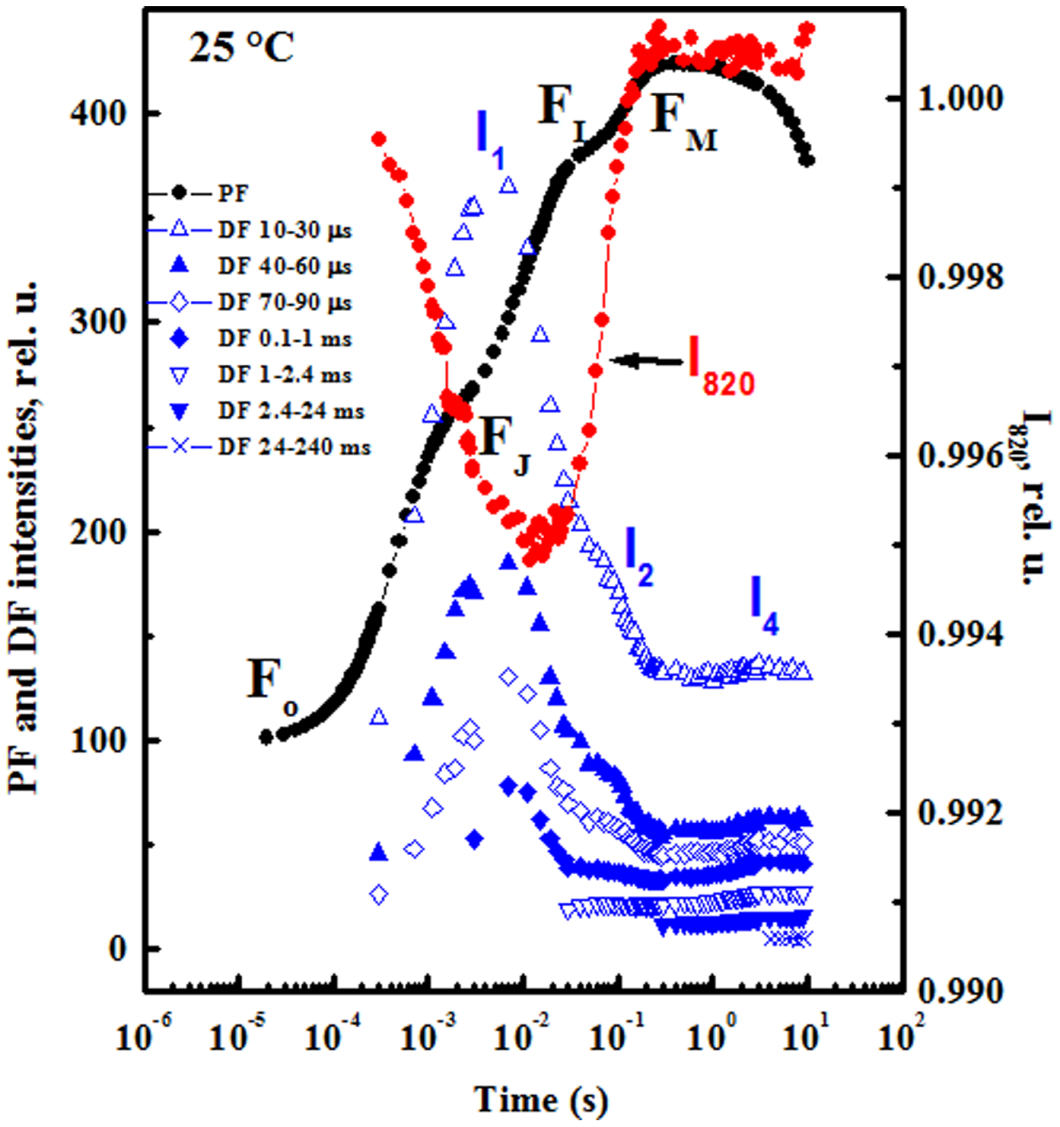
Simultaneous measurements of prompt fluorescence (PF), delayed fluorescence (DF) (left vertical axis) and modulated 820 nm reflection (MR; right vertical axis). Measurements induced by a 10 s pulse of strong red actinic light (627 nm peak, 5000 µmol photons m^−2^ s^−1^) in pea leaves detached from the plants and dark-adapted for 1 h. PF, DF and MR measured simultaneously with M-PEA (see Materials and methods) and plotted on a logarithmic time scale from 20 µs to 10 s (JIP-time). DF vs. delay-time recorded during the dark interruptions of the actinic light (see Materials and methods). The time values present delay-time intervals at which corresponding DF quanta are collected.

The DF induction curves, shown in Figure 1, are averaged DF values collected within different DF delay-time intervals during the dark period after interruptions of the actinic light (the analytical time within each dark interval, during which DF is recorded, was noted as delay-time). Energy level diagram for the PSII-states participating in DF generation has been presented by Grabolle and Dau [13] and Goltsev et al. [32]. The calculated DF values are also presented against the JIP phase of PF [36]. In the DF induction curve, two phases can be observed: the fast one until 200 ms includes the I_1_ and I_2_ peaks, and the slow one until several minutes [8], [32], [37]. Further, included in our study is the I_4_ peak, according to nomenclature of Goltsev and Yordanov [15].

The DF curve measured at 10–30 µs delay-time consists of a fast rise to a peak I_1_ (at 7 ms), a subsequent decrease through I_2_ (at about 100 ms), and I_3_, sometimes found at the end of the fast phase. The origin of I_3_ and the conditions under which it appears are still uncertain. In slow-decaying components, concomitant with the disappearance of the first two maxima (I_1_ and I_2_) in the induction curve, an appearance of the peak I_3_ was observed, and this is mainly due to the slow millisecond components of DF [17]. I_3_ was measured at lower actinic light ∼1200 µmol photons m^−2^ s^−1^ [38]; it was not visible in our experiments at high actinic light (5000 µmol photons m^−2^ s^−1^). The I_4_ level was observed at 5 s and finally a plateau (between 0.5 and 10 s) was observed (Figure 1). Goltsev et al. [39] suggested that the I_1_ maximum is a result of the rise of the transmembrane electrical gradient and of the accumulation of RCs with semi-reduced Q_B_ (Z^+^P680Q_A_Q_B_^−^), while I_2_ was associated with the increase of Z^+^P680Q_A_^−^Q_B_^−^ states during PQ pool reduction. Zaharieva et al. [30] reported that the I_2_ maximum was probably related to the prolonged reopening of PSII RCs by the electron transfer from the reduced Q_B_ to PQ before the reduction of the PQ pool. They suggest that the relative size of this maximum increases with the decrease of the size of the PSII antenna and when the measuring temperature is increased.

In Figure 1, I_1_ is between the J-step towards the I-step of the PF curve and in the oxidation phase of MR measurements and corresponds to the time of decrease of MR (7–10 ms). I_2_ appeared in the range of the I–P phase of the PF and the re-reduction phase of MR measurements. I_4_ appeared during the decline of the PF curve. The three latest DF induction curves (DF vs. JIP-time) the peaks I_1_ and I_2_ disappeared (Figure 1).

## Results

High temperature induced modifications of photosynthetic activity in detached pea leaves were measured with M-PEA after dark treatment for 1 h. Figure 1 shows the kinetics of three signals (PF, DF and MR) obtained by illuminating a leaf-sample with a 10 s pulse of strong red actinic light (627 nm peak, 5000 µmol photons m^−2^ s^−1^) after 40 s incubation at 25°C (control conditions). The induction curves of PF, DF (left vertical axis) and MR (right vertical axis) are plotted on a logarithmic time scale from 20 µs to 10 s.

**Figure 2:**
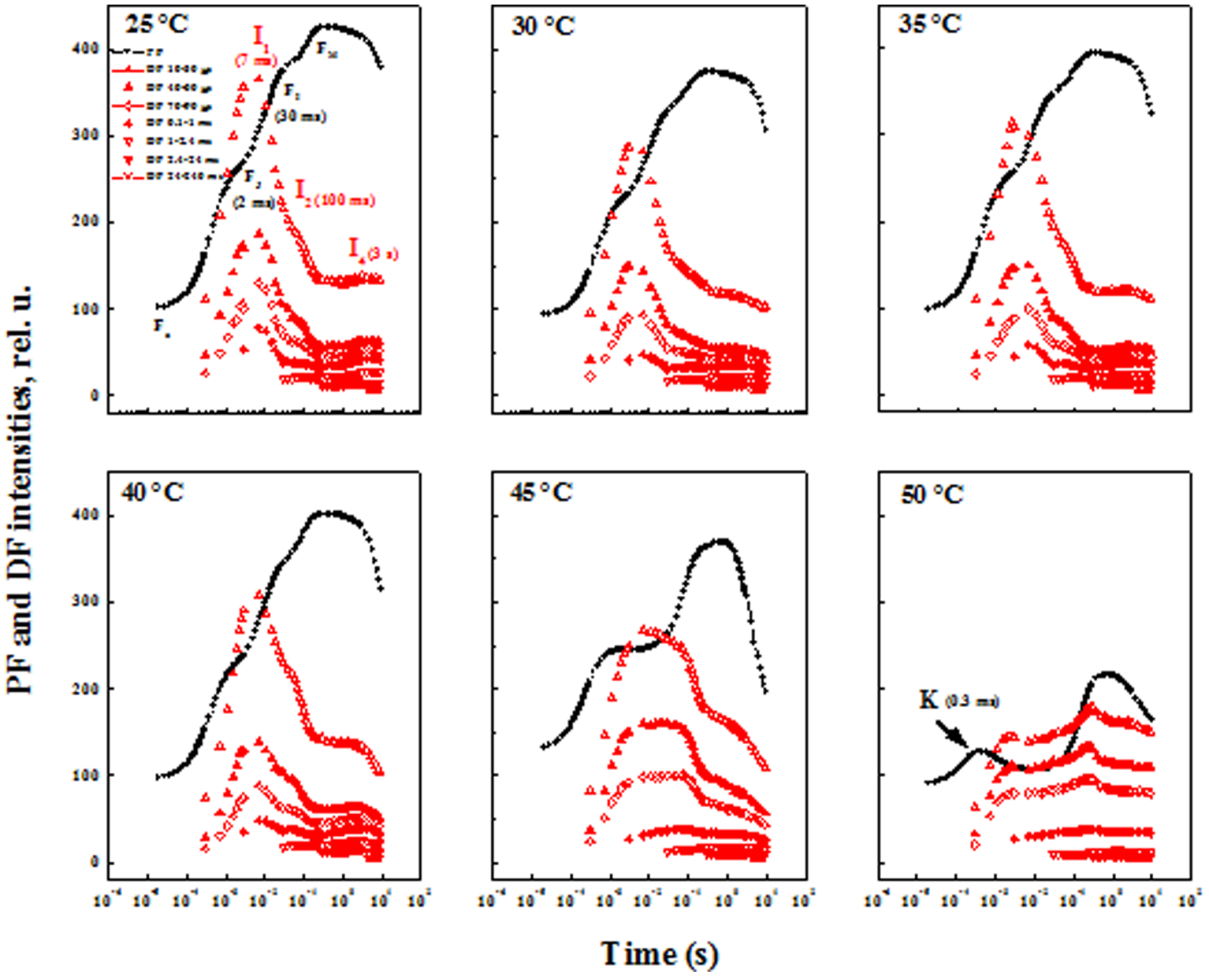
Simultaneous measurements of prompt (PF) and delayed fluorescence (DF) (left vertical axis). Measurements induced by a 10 s pulse of strong red actinic light (627 nm peak, 5000 µmol photons m^−2^ s^−1^) in pea leaves detached from the plants dark-adapted for 1 h and heated to various temperatures (25, 30, 35, 40, 45 and 50°C) for 40 s in darkness.

Figure 2 shows the changes in the shape and intensity of PF and DF transients of pea leaves following 40 s of exposure to different temperatures (25–50°C). We did not observed any change in shape and intensity of the PF and DF curves when the incubation temperature was increased up to 40°C. However the high temperature effect becomes more prominent as the temperature is increased beyond 40°C. After incubation at 45 and 50°C, we observed a decrease in the amplitude of the DF curve and a change in its shape with disappearance of DF decay between the I_1_ and I_2_ peaks. After 50°C incubation, the amplitude of DF curves decreased more than after 45°C incubation, and a decline of I_1_ was observed at 3 ms. After 50°C incubation, the peaks at I_1_ and I_2_ of the DF curve, observed at 7 and 100 ms, disappeared but we saw another peak at 200 ms, which seems to be different from that of I_4_. In the PF curve, the K peak (0.3 ms) appeared clearly after treatment at 50°C and its appearance has been associated with the destruction of the oxygen-evolving-complex (OEC) by heat stress [19], [23].

### Kinetics of Delayed Fluorescence

**Figure 3:**
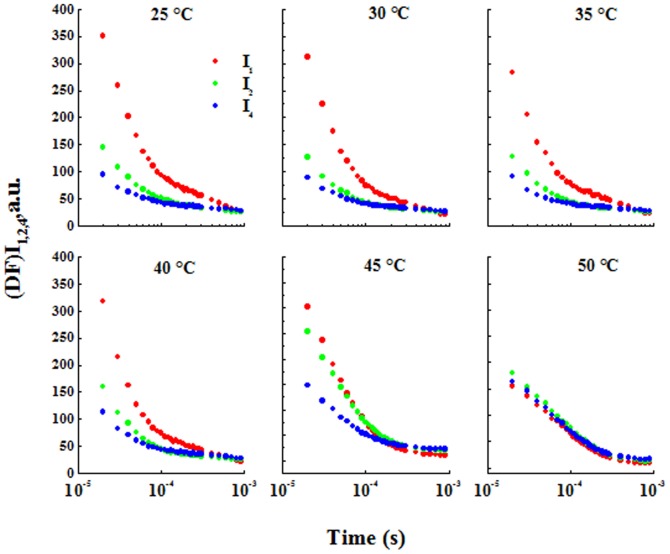
DF decay kinetics at the characteristic maxima. I_1_, I_2_ and I_4_ (at 7, 100 and 5000 ms respectively), indicated by closed circles, open circles and gray circles, respectively.

**Figure 4:**
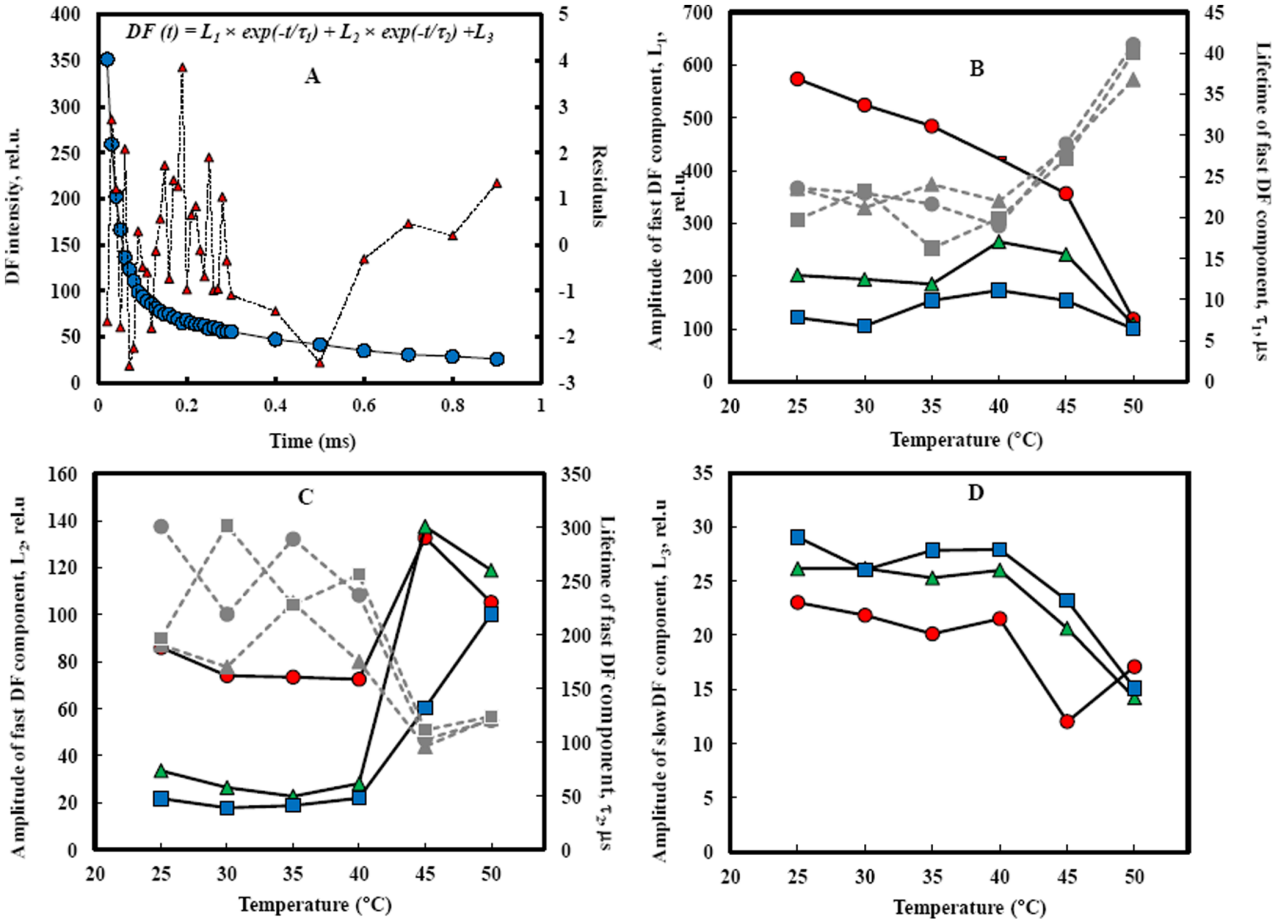
Deconvolution of the data monitored in the time window of 10 to 900 µs. (**A**) Deconvolution was performed by a numerical fit to the formula 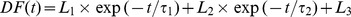 and the residual (triangle symbol). Amplitudes of the kinetic components (open symbols) *L*_1_ (**B**), *L*_2_ (**C**) and *L*_3_ (**D**). *τ*_1_ (**B**) and *τ*_2_ (**C**) (closed symbols) are their lifetimes. I_1_, I_2_ and I_4_ were presented respectively by circles, triangles and squares.

Figure 3 shows DF decay kinetics at I_1_, I_2_ and I_4_ (at 7 ms, 100 ms and 5 s, respectively); here, the DF decay kinetics is presented on a logarithmic time scale, from 0.01 to 0.9 ms (the common range for all three DF decay kinetics). For treatments from 25 to 40°C, the three decay kinetics were different in their average decay rates; this rate was largest for I_1_. However after 45°C incubation, the decay kinetics at I_1_ and I_2_ were the same but still highest compared to the decay kinetics at I_4_. At 50°C, a higher decrease in the two decay kinetics at I_1_ and I_2_ was observed, and I_1_, I_2_ and I_4_ had the same decay kinetics. At microsecond and sub-millisecond time ranges, the DF dark relaxation curve is a polyexponential function. Figure 4A shows a deconvolution of the data monitored in the time window of 10 to 900 µs; it was performed by a numerical fit to the formula 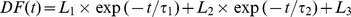, where *L*_1_, *L*_2_ and *L*_3_ are the amplitudes of the kinetic components and *τ*_1_ and *τ*_2_ are their lifetimes [38]. Two components were found in DF decay measured at the beginning of the induction curve (I_1_) in non-treated samples. The fast component has the amplitude *L*_1_ of 573.8±8.2 and lifetime *τ*_1_ of 23.6±0.5 µs. It was followed by a middle, sub ms component, that was characterized by *L*_2_ = 86.0±1.9 and *τ*_2_ = 300.9±23.4 µs. The possible origin of the fast DF component is charge recombination in the PSII reaction center in state ZP680^+^Q_A_^–^ [32], [40]. This DF is considered to be a leakage type luminescence decay; it is a result of separated charge stabilization by re-reduction of *P*680^+^ by Yz. The 35-µs kinetics of 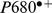
reduction was shown in PSII membrane particles [41], [42], but it seems that it is coupled with additional processes (most likely proton and/or hydrogen transfer) which changes the fluorescence quantum yield [43], [44]. If we propose that all light emitting states (LES) (as well as their dark decay reactions) do not change during induction, we would expect to find that the temperature dependence of the amplitudes of DF components (and their characteristic times), measured at I_1_, I_2_ and I_4_, would be similar, i.e., the temperature dependence of L_1_(I_1_)∼L_1_(I_2_)∼L1(I_4_) and *τ*_1_ (I_1_)∼*τ*_1_ (I_2_)∼*τ*_1_ (I_4_). But the data presented in Figure 4B, C, D show that this is not the case. Although the temperature dependences for *τ*_1_ at times of I_1_, I_2_ and I_4_ are similar, for *τ*_2_ they are similar also at time of I_1_, I_2_ and I_4_ points and amplitudes of DF components measured at I_4_ point showed different temperature dependence as compare to points I_1_ and I_2_ (it showed higher termostability). This fact is valid for L_2_ and L_3_ parameters. It is possible that LESs that are responsible for DF emission are more thermostable after 5–10 s illumination than in the beginning of the induction curve as a result of thylakoid membrane energization or as a result of acidification of the lumenal space. The amplitudes of the fast DF component, measured at the three induction points (I_1_, I_2_ and I_4_), decreased after incubation at temperatures higher than 40°C (Figure 4B). Inhibition of DF was accompanied by slowing down of the DF decay.

The sub-ms (120–200 µs) component [12] in the DF decay curve is suggested to be leakage type luminescence generated from the reaction centers in the 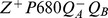 state [39], and the deactivation of these states is a result of the forward reaction of re-oxidation of Q_A_^–^ by Q_B_, which occurs with a rate constant of 2500–5000 s^−1^ [45]. High temperature pre-treatment has minimal effect on the middle component at temperatures below 40°C (Figure 4C). The temperatures 45–50°C accelerated the DF decay (decrease of τ_2_) and activated the DF emission (increased L_2_). The slow DF component is expressed by a constant L_3_. It reflects a sum of sub-millisecond and millisecond DF components. They are results of electron transport from Q_A_ to Q_B_ and from reduced Q_B_ to the plastoquinone pool [32], [39]. Similar to the fast component, the amplitude of the slow component also decreased at two higher temperatures (Figure 4D).

### Relationship between Prompt and Delayed Fluorescence

**Figure 5:**
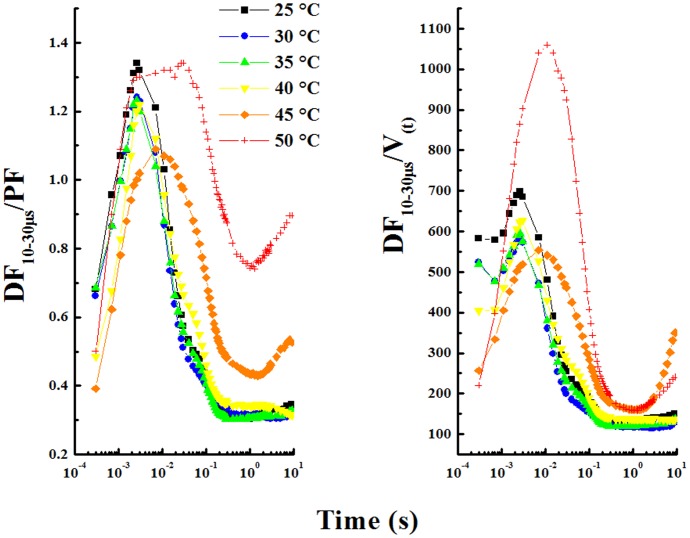
Ratios of delayed fluorescence intensity DF_10–30 µs_/PF and DF_10–30 µs_/V_t_. (V_t_ = (F_t_–F_o_)/(F_M_−F_o_)) measured at 20 µs after the interruption of the actinic illumination in pea leaves detached from the plants dark-adapted for 1 h and heated to various temperatures (25, 30, 35, 40, 45 and 50°C) for 40 s in darkness. V_t_ is the relative variable fluorescence.

Figure 5 shows DF_10–30 µs_/PF, and DF_10–30 µs_/V_(t)_ vs. time, where DF_10–30 µs_ is delayed fluorescence signal at 10–30 µs delay time, and PF represents prompt fluorescence (in arbitrary units) and V_(t)_ is the relative prompt fluorescence ((F_t_-F_o_)/(F_M_-F_o_)). DF/PF expresses the rate of repopulation of excited Chl per absorption [16]. We observe that the shape of (DF_10–30 µs_/PF) curve is different than that of DF_10–30 µs_ curve (see Figure 2). The response to different temperatures after 5 min relaxation at room temperature is practically similar for heated leaves at 25, 30, 35 and 40°C treatment. However I_1_ peak shifts from 7 ms at 25°C to 10 ms and 30 ms respectively after 45 and 50°C treatment. The same observation was made for DF_10–30 µs_/V_(t)_ vs. JIP-time curve, and, in addition, the amplitude of DF after 50°C treatment was found to be strongly higher.

**Figure 6:**
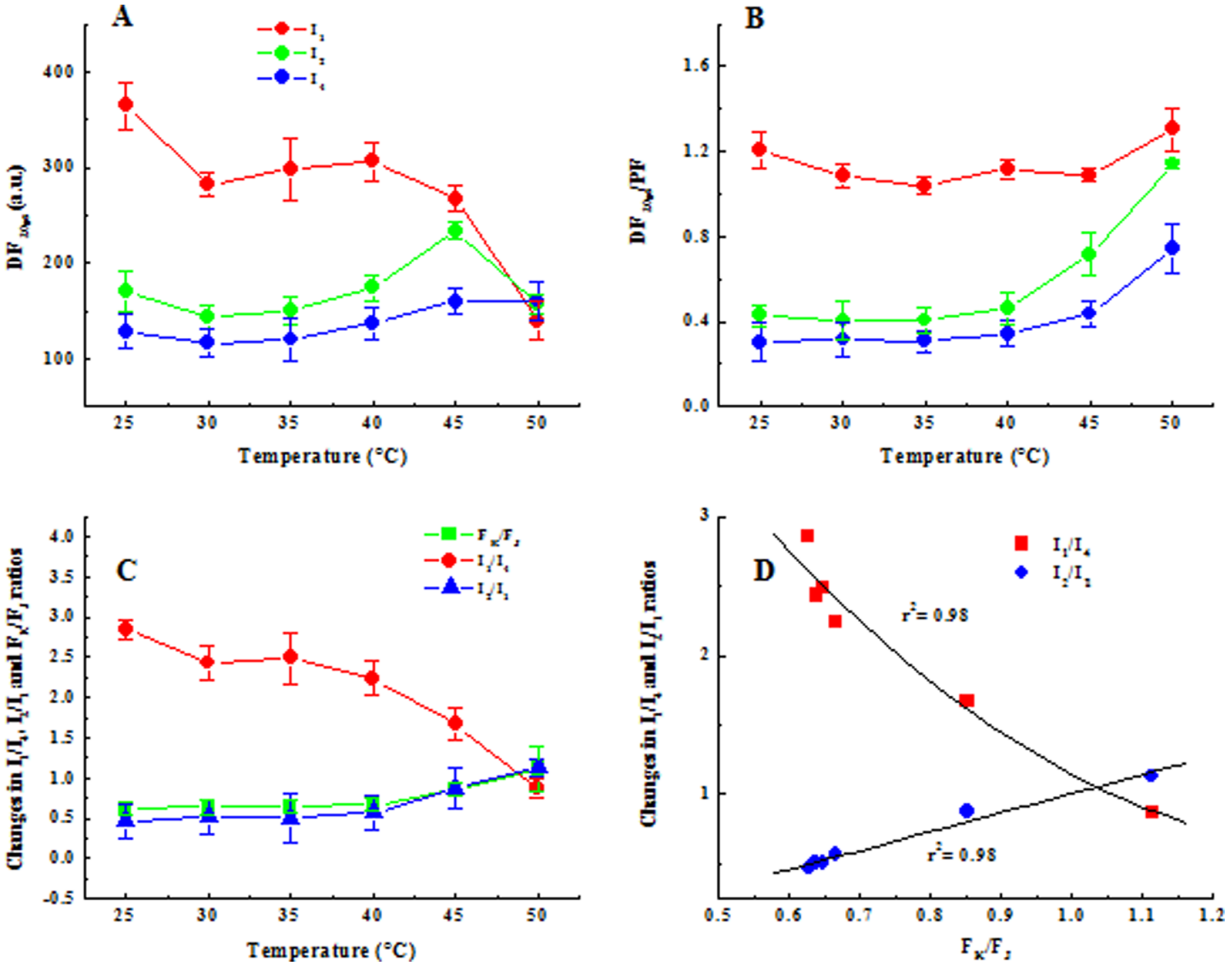
Change of I_1_, I_2_ and I_3_ peaks obtained from DF_10–30 µs_ (**A**) I_1_, I_2_ and I_3_ peaks were plotted vs. temperature treatment. **B.** Change of I_1_, I_2_ and I_3_ peaks obtained from DF_10–30 µs_ normalized to the corresponding value obtained from PF vs. temperature treatment. (**C**) Changes in I_1_/I_4_, I_2_/I_1_ ratios and the amplitude of the K step, expressed as F_K_/F_J_ ratio to different temperature. (**D**) Correlation between F_K_/F_J_ and I_2_/I_1_ and I_1_/I_4_ parameters. The results are shown as the mean with standard deviations.

In Figure 6 A, the I_1_, I_2_ and I_4_ peaks, obtained from DF_10–30 µs_ curves, were plotted vs. temperature treatment. We observed that, upon temperature increase to 40°C, the DF_10–30 µs_ curves decreased in amplitude for I_1_ and increased for I_2_ and I_4_. And at 50°C the I_2_ amplitude decreased. The changes in the amplitude in I_1_/I_4_, I_2_/I_1_ ratio and the K step, expressed as F_K_/F_J_ ratio, after different temperature treatment, are shown in Figure 6B. An increase of F_K_/F_J_ and I_2_/I_1_ ratios was observed and had similar results. On the other hand the I_1_/I_4_ ratio decreased significantly after the incubation temperature was increased. We observe that in the DF/PF curve the shoulders I_2_ and I_4_ increase but are more pronounced in I_2_ (Figure 6C). When we compare these data with F_K_/F_J_, a positive linear correlation with the I_2_/I_1_ ratio is observed (r^2^ = 0.98) and this correlation is exponential with the I_1_/I_4_ ratio (r^2^ = 0.98) (Figure 6D).

### Modulated Reflection at 820 nm

**Figure 7:**
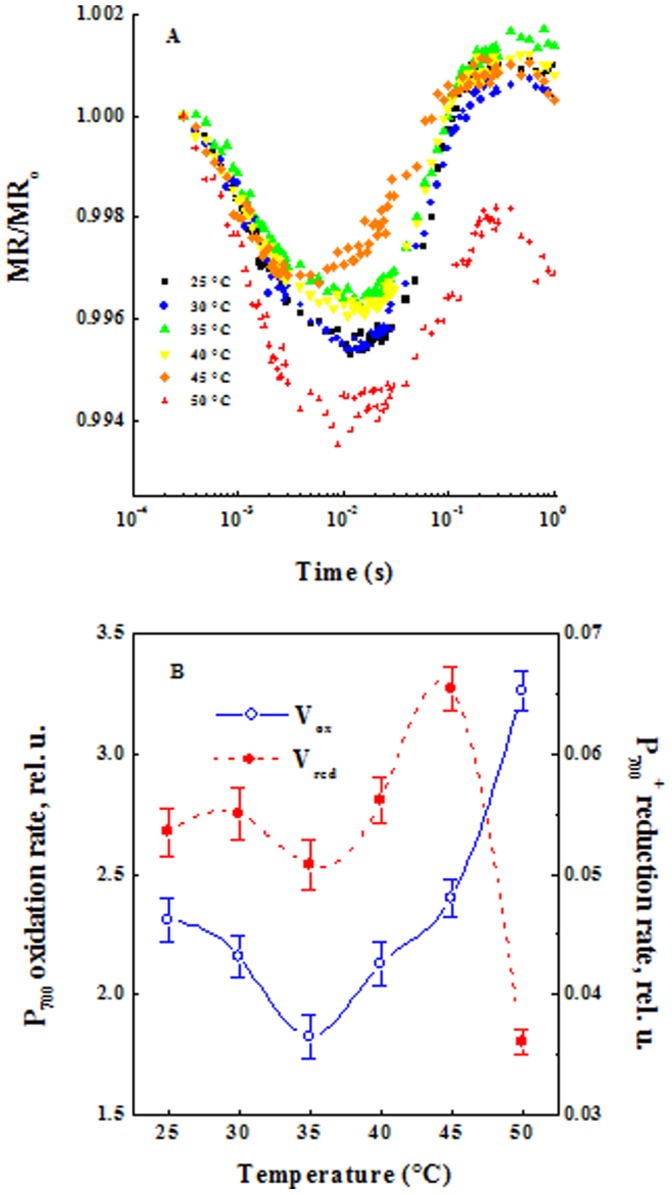
Kinetics of modulated reflection at 820 nm (MR). (**A**) MR induced by red actinic light of 5000 µmol photons m^−2^ s^−1^ in pea leaves detached from the plants and dark-adapted for 1 h and heated to various temperatures (25, 30, 35, 40, 45 and 50°C) for 40 s in darkness. (**B**) Maximal slopes of the kinetics curve of photoinduced MR changes (the rates of P700 and PC oxidation and their followed re-reduction), the results are shown as the mean with standard deviations.

Figure 7A show the kinetics of the normalized modulated reflection at 820 nm (MR) induced by red actinic light of 5000 µmol photons m^−2^ s ^−1^ in heated leaves. Kinetic changes at 820 nm reflect the redox states of P700 and PC. There is an initial oxidation of P700 and PC followed by a re-reduction when electrons arrive from PSII [2]. After 40°C incubation, kinetic changes at 820 nm showed similar re-reduction of P700 and PC and this occurred after 20 ms. After 45°C incubation re-reduction kinetics of P700^+^ and PC^+^ were faster and occurred after 15 ms. On the other hand, after 50°C treatment re-reduction kinetics of P700^+^ and PC^+^ were much slower as observed in [31]. From the maximal slopes of the kinetic curves of photoinduced MR changes, the rates of P700 and PC oxidation and the following re-reduction can be calculated.

The temperature dependences of the values of the slopes are shown in Figure 7B. After 40 s incubation at room temperature, the calculated value of the P700 photooxidation relative rate was of about 2.3 ms^−1^ and for re-reduction –0.054 ms^−1^. The first value reflects the rate of photoinduced electron transfer through PSI and the second – the difference between the rates of electron donation by PSII towards PSI and acceptance of the electrons by PSI. Electron transfers from PSII to PQ, cytochrome (cyt) b6/f complex and PC were faster than those from PSI to Fd. The rate of P700 oxidation was slowed down by 20% after 40 s heating at 35°C and accelerated by 40% after 50°C. The P700^+^ reduction rate was increased by 22% after 45°C heating and inactivated by 33% after 50°C incubation.

## Discussion

PF, DF and MR signals show a distinct temperature response in pea leaves (Figure 2). An increase in the F_K_/F_J_ ratio indicates that heat treatment provokes an inhibition of the donation of electrons by the OEC [46]. The lower electron transport rate through PSII after 45 and 50°C incubation changes DF induction (Figure 2) by showing a decrease in DF yield, which reflects the redox state of the PSII acceptors. Čjánek et al. [47] reported that at temperature above 40°C, the DF signal drops below the level observed at 35°C. Higher temperature of 45°C and 50°C cause a decrease in the peaks yields at I_1_ (7 ms) and I_2_ (100 ms), disappearance of DF decay from I_1_ to I_2_ and the disappearance of the I_1_ peak at 50°C. The disappearance of DF decay between the I_1_ and I_2_ peaks was in parallel to the disappearance of the J-I phase from the PF curve. In this phase (J-I) a progressive reduction of the plastoquinone (PQ) pool occurs [3] (see [4] for a discussion about the J-I-P thermal phase). The block of PSII reaction centers as well as the electron flow from the reduced Q_A_ to Q_B_ has been reported to be damaged at higher temperatures [31]. Therefore, I_1_ to I_2_ decay might be related to the redox states of the PSII reaction center when the electron transfer from reduced Q_B_ to PQ begins.

DF decay kinetics is composed of several components denoted as LES [32]. The increase and then the decrease of I_2_ (measured at 100 ms) may be related to the oxidation-reduction of the PQ-pool, which may be first activated by the increased temperature (due to the increased lipid fluidity of the thylakoid membranes that could lead to a higher diffusion rate of PQ molecules), and then it may be inactivated, probably because of protein disorganization at higher temperatures. The maximum I_4_ occurs in parallel with a decrease of the PF intensity. We noted here that I_4_ was less affected by heat treatment although after 50°C incubation its increase might be attributed to Q_A_^−^ accumulation.

We observed that the DF curves are more affected than PF curves at temperatures of 25, 30, 35 and 40°C. This may mean that DF curves are more thermosensitive than PF curves. Indeed, at these temperatures, we observed a decrease in the amplitude of the DF curve and change of I_1_, I_2_ and I_3_ peaks obtained from DF_10–30 µs_, while minor significant changes in PF curves were monitored. The relative I_2_ in the fast DF phase increased and the relative DF induction increased in the slow phase. The increase of relative DF in the slow phase might be related to the activation of the Calvin-Benson cycle (Figure 5). These observations have been reported by Zaharieva et al [30] when leaves of wild type and mutants of Arabidopsis were exposed to increased temperature to 45°C.

A decrease in F_M_ (from 30°C) and increase in F_o_ (45°C) were observed. Increase of F_o_ has been suggested to be due to release of LHC II from the PS II complex and inactivation of PS II photochemical reaction [48], or an inhibition of electron flow from reduced Q_A_ to Q_B_ [49], [50]. Yamane et al. [51], [52] have also reported that the increase of F_o_ was also due to irreversible dissociation of LHC II from the PS II complex and partly reversible inactivation of PS II in spinach and rice. The decrease in the fluorescence F_M_ level seemed to be related to denaturation of chlorophyll-proteins [51].

It is known that heat treatment leads to an inhibition of the donation of electrons by OEC, by a loss of the manganese cluster which leads to changes in the structure and function of PSII [18], [22], [23]. Indeed, the K peak observed in the PF curve after 50°C treatment reflects this change by partial Q_A_ reduction, which is due to a stable charge separation resulting from the donation of one electron by tyrozine Z [23], [31]. Therefore the absence of electron donors would lead to the accumulation of P680^+^. The decline of I_1_ to ∼3 ms observed in kinetics of fast DF components (measured at 10–30 µs delay-time) at 50°C occurred in parallel to the appearance of the K peak in the PF curve. We can assume that this decline might be related to the redox states of the PSII reaction center when the K step appeared in PF curve or in other words when OEC was destroyed. However, this decline can be related to the accumulation of P680^+^ and DF emission after recombination of LES P680^+^Q_A_^–^. The absorption of only one light quantum is enough to form such a state. This is the reason for the short time for the appearance of this maximum. In dark–adapted state, samples start in 75% S_1_ and 25% S_o_ [53] and, therefore, the low luminescence state S_1_Z^+^Q_A_^–^Q_B_ is formed before it is transformed to the high luminescence state S_3_Z^+^Q_A_^−^Q_B_; to complete this transformation, every RC needs to absorb two quanta for storing two electrons before donating them to the plastoquinone pool, PQ [54]. Lazár [45] reported that the rise of fluorescence to the J step in the OJIP transient is much more suppressed when all OEC is initially in the S_2_ or S_3_ states and a new step appears in the OJIP transient located at the position of the K step. Using thermoluminescence (TL) method, an AG band peaking between 40 and 50°C has been observed [55], [56]. This AG band emission is stimulated by high temperature, and corresponds to a heat-induced stimulation of electron transfer from stromal reductants to PSII centers that are initially in the non-recombining state S_2_/_3_Q_B_ and are progressively converted to light-emitting S_2_/_3_Q_B_^−^ states [55], [56].

Figure 6C shows a linear correlation between the F_K_/F_J_ and I_2_/I_1_ ratios. Indeed, this parameter F_K_/F_J_ was introduced by Srivastava and Strasser [46] in describing heated pea leaves. F_K_/F_J_ has been also introduced to monitor electron donation limitations on the donor side of PSII in barley varieties differing in their drought tolerance [57]. The I_2_/I_1_ ratio has been used as an indication for electron flow acceleration through the PSII acceptor side [15]. These two parameters (F_K_/F_J_ and I_2_/I_1_ ratios) might indicate the limitation of electron donation on the donor side of PSII and represent a quantitative measure for the inactivation of the PSII donor side. Therefore, they might be excellent parameters for monitoring heat stress effects on PSII.

Acceleration of P700^+^ and PC^+^ re-reduction was induced by 45°C treatment but after 50°C its reduction was slower, indicating PSI inhibition. However, many investigators have found that moderate heat stress increases PSI activity, often at the expense of the redox status of the stroma [57], [58]. We note that PSI accelerates the oxidation rate at 50°C incubation with 80% as compared to that at 35°C (Figure 7B).
